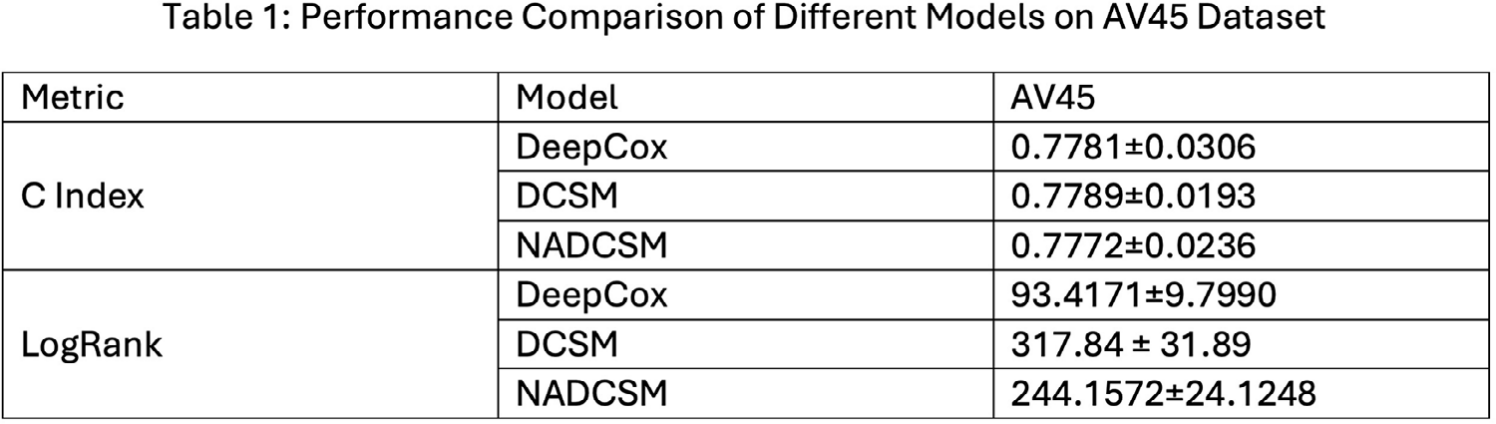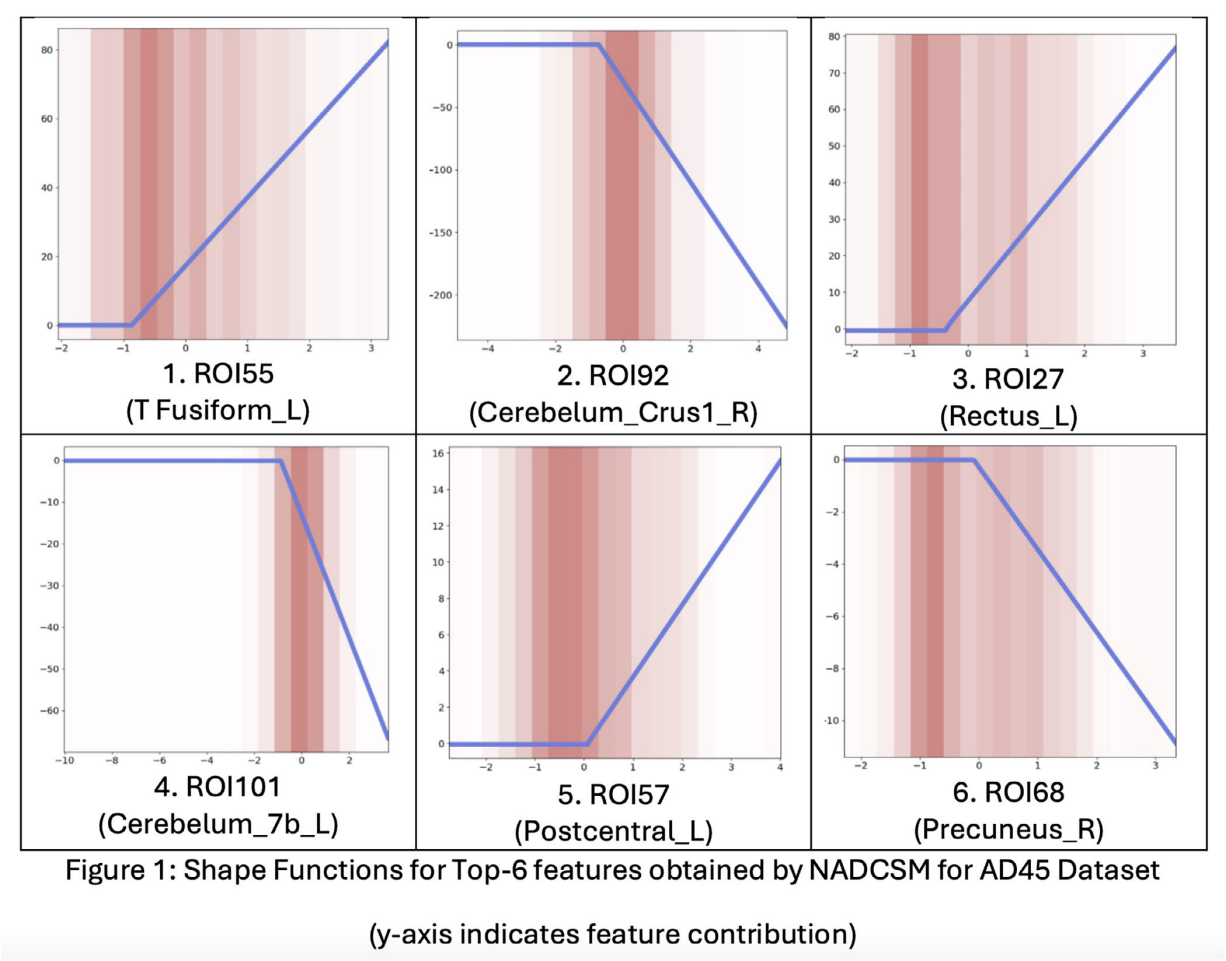# Neural Additive Deep Clustering Survival Machines: An Interpretable Approach for Alzheimer's Disease and Related Dementias

**DOI:** 10.1002/alz70855_107083

**Published:** 2025-12-25

**Authors:** Kazi Noshin, Bojian Hou, Mary Regina Bolan, Li Shen, Aidong Zhang

**Affiliations:** ^1^ University of Virginia, Charlottesville, VA, USA; ^2^ University of Pennsylvania, Philadelphia, PA, USA; ^3^ Saint Vincent College, Latrobe, PA, USA; ^4^ Department of Biostatistics, Epidemiology, & Informatics, University of Pennsylvania, Philadelphia, PA, USA

## Abstract

**Background:**

In Alzheimer's Disease research, identifying brain regions that influence disease progression is crucial for understanding pathogenesis and developing targeted therapies. Deep learning models have advanced survival analysis in Alzheimer's Disease research, but their “black box” nature limits clinical utility. We developed Neural Additive Deep Clustering Survival Machines (NADCSM), which leverages Neural Additive Models to provide interpretable insights into brain region contributions while maintaining competitive predictive performance. This approach aims to bridge the gap between model performance and clinical utility, potentially accelerating biomarker discovery and therapeutic development.

**Method:**

The genotyping, demographic, and imaging data used in our experiments are sourced from the Alzheimer's Disease Neuroimaging Initiative (ADNI) database. We utilized ADNI's AV45 Florbetapir PET imaging data to track MCI and early AD progression. This modality is particularly valuable for tracking the progression of mild cognitive impairment (MCI) and early Alzheimer's disease (AD). Our NADCSM framework models survival times using Weibull distributions and enhances interpretability through Neural Additive Models (NAMs)., where each input feature is processed through multiple univariate shape functions parameterized by multilayer perceptions (MLPs). Performance was evaluated using Concordance Index (C Index) for predictive ability and LogRank statistic for survival curve separation and clustering quality.

**Result:**

Table 1 compares the performance of DeepCox, DCSM, and NADCSM (ours) on the AV45 dataset using C Index and LogRank metrics. DCSM achieves the highest C Index (0.7789 ± 0.0193), followed by DeepCox (0.7781 ± 0.0306) and NADCSM (0.7772±0.0236), demonstrating strong predictive accuracy. For LogRank, DCSM leads (317.84 ± 31.89), followed by NADCSM (317.84 ± 31.89) and DeepCox (93.4171±9.7990), indicating NADCSM's competitive performance with enhanced interpretability.

Figure 1 shows shape functions for the top six brain ROIs identified by NADCSM, including Fusiform (Left), Cerebelum_Crus (Right), and others. The smooth curves capture the relationships between regional amyloid burden and AD progression, highlighting key survival predictors.

**Conclusion:**

Our NADCSM framework provides an interpretable risk prediction approach, uncovering significant features and explaining their effects on Alzheimer's and Related Dementias (ADRD) progression. By enhancing transparency, this study can advance precision medicine and improve understanding of ADRD‐related challenges.